# Ameliorating Amyloid-β Fibrils Triggered Inflammation *via* Curcumin-Loaded Polymeric Nanoconstructs

**DOI:** 10.3389/fimmu.2017.01411

**Published:** 2017-10-31

**Authors:** Andrea Ameruoso, Roberto Palomba, Anna Lisa Palange, Antonio Cervadoro, Aeju Lee, Daniele Di Mascolo, Paolo Decuzzi

**Affiliations:** ^1^Laboratory of Nanotechnology for Precision Medicine, Fondazione Istituto Italiano di Tecnologia, Genoa, Italy; ^2^International Research Organization for Advanced Science and Technology (IROAST), Kumamoto University, Kumamoto, Kumamoto Prefecture, Japan

**Keywords:** nanoparticle, inflammation, systemic delivery, neurodegenerative diseases, macrophage activation

## Abstract

Inflammation is a common hallmark in several diseases, including atherosclerosis, cancer, obesity, and neurodegeneration. In Alzheimer’s disease (AD), growing evidence directly correlates neuronal damage with inflammation of myeloid brain cells, such as microglia. Here, polymeric nanoparticles were engineered and characterized for the delivery of anti-inflammatory molecules to macrophages stimulated via direct incubation with amyloid-β fibers. 200 nm spherical polymeric nanoconstructs (SPNs) and 1,000 nm discoidal polymeric nanoconstructs (DPNs) were synthesized using poly(lactic-*co*-glycolic acid) (PLGA), polyethylene glycol (PEG), and lipid chains as building blocks. First, the internalization propensity in macrophages of both nanoparticles was assessed *via* cytofluorimetric and confocal microscopy analyses, demonstrating that SPNs are by far more rapidly taken up as compared to DPNs (99.6 ± 0.11 vs 14.4 ± 0.06%, within 24 h). Then, Curcumin-loaded SPNs (Curc-SPNs) were realized by encapsulating Curcumin, a natural anti-inflammatory molecule, within the PLGA core of SPNs. Finally, Curc-SPNs were shown to diminish up to 6.5-fold the production of pro-inflammatory cytokines—IL-1β; IL-6, and TNF-α—in macrophages stimulated *via* amyloid-β fibers. Although more sophisticated *in vitro* models and systematic analyses on the blood–brain barrier permeability are critically needed, these findings hold potential in the development of nanoparticles for modulating inflammation in AD.

## Introduction

Inflammation is a defense response to external pathogens and other insults which is precisely orchestrated by our immune system. However, under certain conditions, inflammatory processes could become detrimental and lead to severe pathological states (1). This is the case of atherosclerosis where monocyte infiltration into the vessel walls and maturation into macrophages are key events in the formation and progression of vascular plaques (2). Inflammation and immune cells play a role in cancer too where tumor-associated macrophages could protect and sustain the growth of malignant cells (3). Moreover, in obesity, macrophage infiltration in adipose tissue causes local and systemic inflammation eventually leading to insulin resistance (4). Similarly, in Alzheimer’s disease (AD), mounting evidence indicates that macrophages of the central nervous system—microglia—contribute to the onset of the disease and sustain neurotoxicity (5, 6). While under physiological conditions, microglia serve as immune surveilling cells, upon injury or immune stimuli activation, microglia secrete pro-inflammatory cytokines that are eventually responsible of neuronal death. Following the amyloid cascade hypothesis, microglia activation is triggered in Alzheimer’s disease by amyloid-β plaques (Aβs), resulting from the extracellular accumulation of amyloid-β peptides, and neurofibrillary tangles, deriving from the clustering of the microtubule-associated protein tau. Over a sufficiently long time, this is responsible of chronic brain inflammation and production of pro-inflammatory cytokines, such as IL-1β, IL-6, TNF-α, and many others (7).

Nanomedicines are slowly but surely accessing clinical practice for the treatment of deadly diseases, so far primarily involving cancer and cardiovascular (8, 9). Over 40 liposomal and polymeric nanoparticles are currently undergoing clinical investigation and a few nano-based products are already routinely used by oncologists (liposomal doxorubicin and albumin-bound paclitaxel). Over single therapeutic agents, nanomedicines can carry and deliver multiple drug molecules to diseased sites following specific release profiles; protect the payload from enzymatic degradations and enhance bioavailability; provide useful information at the cellular and tissue scales for designing patient-specific therapeutic interventions. Furthermore, the size, shape, surface properties, and mechanical stiffness of nanomedicines can be often precisely tailored during the fabrication process to enhance accumulation at the biological target and mitigate adverse effects deriving by off-targeting (10–15). Importantly, properly designed nanoparticles can be rapidly taken up by activated macrophages residing in different vascular districts and tissues. This gives the opportunity of efficiently using nanoparticles to deliver directly into activated macrophages anti-inflammatory agents, possibly modulating both locally and systemically the inflammatory state. The authors and other groups have loaded nanoparticles with a variety of anti-inflammatory molecules, starting with the natural, broad-spectrum molecule Curcumin and moving to drugs with more specific sub-cellular targets as diclofenac (16–18). Note that both Curcumin and diclofenac are hydrophobic, exhibit a poor bioavailability and their specific, systemic administration can be largely improved *via* encapsulation into nanoparticles.

This work aims at selecting nanoparticles for the specific delivery of Curcumin to macrophages which have been activated by incubation with amyloid-β fibrils. First, two different nanoparticle configurations were considered, namely spherical polymeric nanoconstruct (SPNs) with a characteristic size of about 200 nm and discoidal polymeric nanoconstructs (DPNs) with a diameter of 1,000 nm and height of 400 nm (15, 17). Both nanoparticles were realized with biodegradable and biocompatible polymers—poly(lactic-*co*-glycolic acid) (PLGA), polyethylene glycol (PEG)—mixed with lipid chains. Then, the most effective configuration was selected based on macrophage internalization assays involving cytofluorimetric and confocal microscopy analyses. Finally, the selected configuration was loaded with Curcumin and delivered to macrophages, in the presence of amyloid-β fibrils, for assessing IL-1β; IL-6 and TNF-α production.

## Materials and Methods

### Materials

Poly(lactic-*co*-glycolic acid) (50:50, Carboxy-terminated, MW 38,000–54,000 Da) was purchased from Sigma Aldrich (St. Louis, MO, USA). 1,2-dipalmitoyl-sn-glycero-3-phosphocholine (DPPC) and 1,2-distearoyl-sn-glycero-3-phosphoethanolamine-*N*-[Carboxy(Polyethylene Glycol)-2000] (DSPE-PEG) were obtained from Avanti Polar Lipids (Alabaster, Alabama). Curcumin (95% total curcuminoid content) was purchased from Alfa Aesar. Chloroform, Acetonitrile and other solvents were obtained from Sigma Aldrich.

### Nanoparticle Synthesis and Characterization

Spherical polymeric nanoconstructs (SPNs) were synthesized by employing an emulsion/solvent evaporation technique (17). DSPE-PEG was dissolved in a 4% ethanol solution to a final volume of 3 ml to obtain the aqueous phase, whereas 1 mg of PLGA and an appropriate quantity of DPPC were dissolved in chloroform to create the oil phase. A v/v ratio of 6:1 between the aqueous and organic phase, a lipids/polymer w/w ratio of 20% and a DPPC/DSPE-PEG molar ratio of 7.5:2.5 were used. Then, the oil phase was added in a dropwise manner to the aqueous solution under ultrasonication at 60% amplitude (Q125 sonicator, Q-Sonica). The resulting emulsion was then gently stirred at room temperature and in a reduced pressure environment for 4 h to allow solvent evaporation. Finally, nanoparticles were washed with water by centrifugation using Amicon Ultra-4, Centrifugal Filter 10,000 Da (Millipore) at 3,500 rpm for 8 min for three times to remove any possible debris obtained in the synthesis process. SPN size and surface zeta potential were estimated by dynamic light scattering (DLS) (Malvern Zetasizer, ZEN 3600). To this end, nanoparticles solution was centrifuged at 12,000 rpm for 20 min and the pellet was resuspended in 1 ml of Milli-Q water; then, 20 µl were diluted in 1 ml of Milli-Q water and the resulting solution was transferred into a folded capillary cell (Malvern). The Smoluchowski model was used to calculate zeta potential values. For scanning electron microscopy (SEM) analysis, the SPN solution was dropped directly onto a polished silicon wafer. After drying, samples were sputter-coated with platinum prior to imaging, to enhance polymer contrast. The stability of the nanoparticles was evaluated over a period of 9 days. Nanoparticles were suspended in 1 ml of Milli-Q water and kept at 37°C for the whole time span. At various time points (namely at days 1, 2, 3, 5, and 9), a DLS analysis on each sample was performed as described above. For each characterization study, the number of analyzed samples was 3.

Discoidal polymeric nanoconstructs were synthesized by employing a top-down fabrication process described in details in our previous works (14, 15). Briefly, this fabrication approach involves the use of electron beam lithography (EBL) to fabricate a silicon master template presenting an array of cylindrical holes with a fixed diameter (1,000 nm) and height (400 nm). This pattern is then replicated into PDMS and subsequently PVA templates, by using soft lithography techniques. Once the holes of the sacrificial template (PVA) are filled with the polymeric mixture composed by PLGA and PEG, the PVA is dissolved in water to collect the resulting particles. To perform internalization experiments, lipid Rhodamine was added to the polymeric mixture composing DPNs. DPN physical chemical characterization was performed through Multisizer (Beckman Coulter) to calculate DPN concentration and size distribution profile, and DLS to estimate the zeta potential. The samples for transmission electron microscopy (TEM) were prepared by the drop casting method over copper grid. The samples were negatively stained for 10 min with 2% (w/v) uranyl acetate aqueous solution, and then washed twice with distilled water and dried before imaging. The stability of the DPNs was evaluated as described above for the SPNs (*n* = 3).

### Cell Cultures

Raw 264.7 cells were purchased from the American Type Culture Collection (ATCC, Rockville, MD, USA) and maintained in Dulbecco’s Modified Eagle’s Medium high-glucose (DMEM) (Euroclone) supplemented with 10% fetal bovine serum (ATCC) and 1% penicillin/streptomycin. Cells were grown at 37°C in an 80% humidified atmosphere of 5% CO_2_.

### Internalization Experiments

2 × 10^5^ RAW 264.7 cells were seeded into a 12 well plate. Cells were treated with SPNs (1.5 µg/ml), Fluoresbrite^®^ Carboxyl NYO Carboxylate Microspheres 0.20 µm (Polyscience) (30 Particles per cell), and DPNs (10 particles per cell). 24 h later cells were harvested in phenol red free DMEM (Lonza) and analyzed by flow cytometry using BD FACS Aria (Beckton Dickinson). 2 × 10^5^ events per sample were analyzed, and experiments were run in triplicate. For microscopy analyses, 1 × 10^4^ RAW 264.7 cells were seeded into a Lab-Tek II Chambered Coverglass (Thermo Fisher). Cells were treated following the same condition described above. Images were acquired using A1 + Nikon confocal microscope system (Nikon). Statistic was performed analyzing four images per each group and performed in triplicate.

### Drug Loading (DL) and Release

Curcumin-loaded SPNs (Curc-SPNs) were synthesized by employing the same emulsion/solvent evaporation technique described above. Simply, the 200 µg of Curcumin was dissolved in chloroform and added to the oil phase per each milligram of PLGA. To estimate loading and encapsulation efficiency (EE), SPNs were resuspended in 1 ml of water and freeze-dried. After lyophilization, a known amount of particles was dissolved in acetonitrile to free the entrapped drug. The absorbance of Curcumin at 430 nm, with a baseline wavelength of 650 nm, was measured and used to calculate the amount of molecule. Each sample was evaluated in triplicate. Loading efficiency was expressed as the weight percentage of drug mass with respect to the total mass of the nanoparticles, whereas EE was expressed as the weight percentage of entrapped drug mass as compared to the initial drug input. The *in vitro* release of Curcumin was evaluated under physiological conditions in PBS at 37°C and pH 7.4 up to 72 h. 200 µl of nanoparticle solution at 10 µM Curcumin were transferred into Slide-A-Lyzer MINI dialysis cups with a molecular cutoff of 10 kDa (Thermo Scientific, Rockford, IL, USA) and dialyzed against 4 l of PBS. At each time point, three replicates were retrieved and analyzed. Quantification of the amount of drug released was obtained through a spectrophotometric measurement, using a method akin to the one employed in assessing the loading efficiency. The content of each cup was collected and centrifuged at 12,000 rpm for 20 min, and the resulting pellet of nanoparticles was dissolved in acetonitrile. Then, for each sample, the absorbance at 430 nm with a baseline wavelength of 650 nm was measured. Results for each time point are expressed as a percentage with respect to the initial time point.

### *In Vitro* Production of Amyloid-β Fibrils

Amyloid-β (Aβ) peptides (Aβ 1–42) (MW 4415.26, Sigma Aldrich) were dissolved by briefly vortexing in a 0,02% ammonia solution at a concentration of 1 mM at 4°C and stored at −80°C. Formation of Aβ fibrils was obtained through a polymerization reaction conducted as described by Ono and Hasegawa: Aβ peptides were dissolved in 50 mM phosphate buffer (pH 7.4, 100 mM NaCl) to a final concentration of 25 µM and to a final volume of 950 µl and incubated for 6 and 16 h at 37°C. The reaction was stopped by storing the samples at 4°C. Aβ fibrils were visualized *via* TEM. 4 µl of fibril solution were cast on a carbon-coated copper grid and positively stained for 10 min with a 2% (w/v) uranyl acetate aqueous solution. Samples were then washed with distilled water and dried before imaging.

### Cell Viability Assay

Dead Cell Apoptosis Kit with Annexin V FITC and PI (ThermoFisher) was used to initially detect any apoptotic effect of PLGA, as main constituent of our particles, both SPNs and DPNs. Cells (2 × 10^5^) were seeded and, after reaching confluency, were treated with empty nanoparticles, at three different concentrations, namely 0.05, 1.5, and 15 µg/ml of PLGA. After 12 h, cells were detached from the plates and stained using the aforementioned kit. Then FACS analyses were performed. An MTT (3-(4,5-Dimethylthiazol-2-yl)-2,5-Diphenyltetrazolium Bromide) proliferation assay (Sigma Aldrich) was used to evaluate the cytotoxicity of free Curcumin, empty nanoparticles, and Curcumin-loaded nanoparticles. Cells were seeded at a density of 5 × 10^3^cells per well in 96 well plates and cultured for 24 h. Free Curcumin was suspended in DMSO and diluted to various concentrations with complete cell media. DMSO was always kept at a final concentration below 0.1% v/v. Empty SPNs and Curcumin-SPNs were resuspended in complete media at various concentrations. These solutions were used to treat cells. After 24 h, the medium was removed and the MTT working solution was added according to the manufacturer’s instructions. After 4 h, medium was removed and DMSO was added to each well to solubilize the purple precipitates. Upon complete solubilization, the absorbance at 570 nm was measured for each sample. In all groups, five replicates were analyzed per each of the used concentration. Data are expressed as the percentage of viable cells with respect to controls.

### Real-time RT-PCR

Raw 264.7 macrophages were seeded at a density of 2 × 10^5^ cells/well in 6-well plates, containing 2 ml of culture media. Cells were pre-treated for 5 h with 10 µM free Curcumin or Curc-SPNs and then exposed to 2 µM Aβ fibrils and 100 ng/ml LPS (Sigma Aldrich). Cells were also exposed to empty SPNs, Aβ fibrils and LPS without previous treatment with Curc-SPNs. After 6 h, total RNA was extracted using RNeasy Plus Mini Kit (Qiagen) according to the manufacturer’s instructions and quantified using Nanodrop 2000 UV-Vis Spectrophotometer (Thermo Scientific). Real Time RT-PCR were carried out using a *Power* SYBR Green RNA-to-C_T_ *1-Step* Kit (Applied Biosystems). The reactions were performed in a final volume of 20 µl of the following reaction mixture: 2X *Power* SYBR Green RT-PCR Mix, 200 nM respective primer pairs, 125X RT Enzyme Mix, 100 ng of RNA template for retrotranscription and amplification of TNF-α, IL-1β, or IL-6 gene product. GAPDH was used as housekeeping gene. Oligonucleotide primer pairs were as follows: for GAPDH, 5′-GAACATCATCCCTGCATCCA-3′ and 5′-CCAGTGAGCTTCCCGTTCA-3′; for TNF-α, 5′-GGTGCCTATGTCTCAGCCTCTT-3′ and 5′-GCCATAGAACTGATGAGAGGGAG-3′; for IL-1β, 5′-TGGACCTTCCAGGATGAGGACA-3′ and 5′-GTTCATCTCGGAGCCTGTAGTG-3′; for IL-6, 5′-TACCACTTCACAAGTCGGAGGC-3′ and 5′-CTGCAAGTGCATCATCGTTGTTC-3′. The fold change in gene expression was evaluated by ΔΔ^Ct^ method, relative to the control. All experimental groups were tested in triplicate.

### Statistical Analysis

Statistical analysis of significance was performed using ANOVA, after that equal-variance assumption was confirmed, using the robust Brown–Forsythe Levene-type test for homogeneity of variance. Multiple comparisons were performed using, as *post hoc* test, the Tukey’s honestly significant difference (HSD) test. Comparisons with a *p*-value lower or equal to 0.05 was considered statistically significant different with respect to control. Data are presented as mean ± SD.

## Results

### Synthesis and Physico-Chemical Characterization of Spherical and DPNs

**Figure 1:**
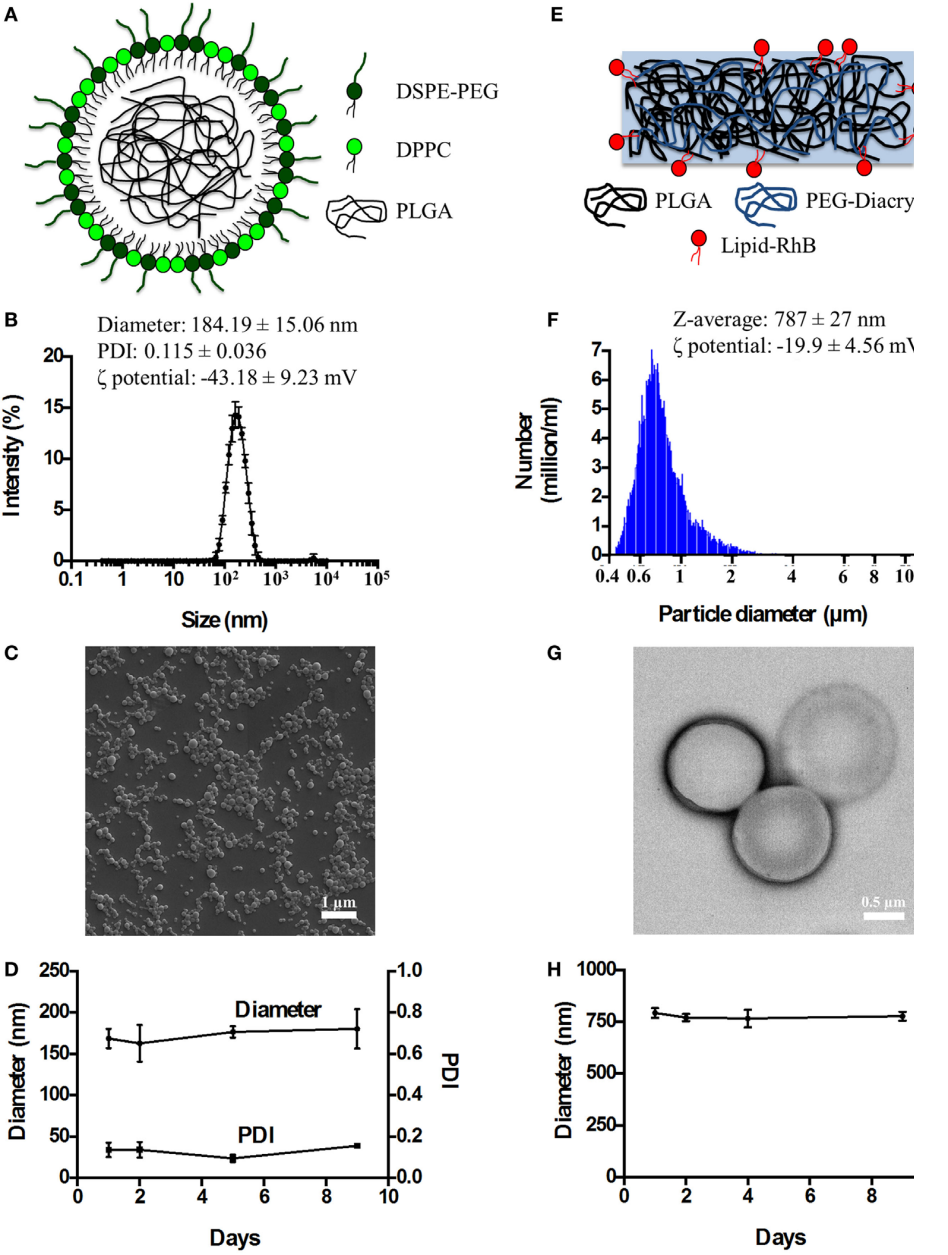
The physico-chemical characterization of spherical and discoidal polymeric nanoconstructs. **(A)** Schematic representation of SPNs synthetized *via* emulsion/solvent evaporation method comprising a hydrophobic [poly(lactic-co-glycolic acid) (PLGA)] core stabilized by an external lipid monolayer (a mixture of DPPC and DSPE-PEG-COOH). **(B)** Size distribution of SPNs *via* dynamic light scattering analysis (*n* = 3). **(C)** Scanning electron microscopy image of SPNs. **(D)** Colloidal stability of SPNs in de-ionized (DI) water (*n* = 3). **(E)** Schematic representation of DPNs synthetized *via* a top-down approach and resulting by the crosslinking of PLGA and poly(ethylene glycol) diacrylate (PEG-DA) chains. **(F)** Size distribution of DPNs *via* Multisizer analysis (*n* = 3). **(G)** Transmission electron microscopy image of DPNs. **(H)** Colloidal stability of DPNs in DI water (*n* = 3).

Spherical Polymeric Nanoconstructs (SPNs) were synthetized *via* an emulsion/solvent evaporation technique, as detailed in the Section “Materials and Methods” and in previous reports by the authors and other scientists (16, 17, 19). As schematically depicted in Figure 1A, SPNs exhibit a hydrophobic polymeric core made out of PLGA which is stabilized externally by a lipid monolayer comprising a mixture of dipalmitoyl-sn-glycero-3-phosphocholine (DPPC) and 1,2-distearoyl-sn-glycero-3-phosphoethanolamine-*N*-[amino(polyethylene glycol)-2000] with a carboxylic termination (DSPE-PEG-COOH). Following synthesis, SPNs were characterized for their physico-chemical properties. Specifically, SPN geometry (size and shape) was analyzed *via* DLS and SEM. The SPN hydrodynamic size in de-ionized (DI) water resulted of 184.19 ± 15.06 nm, with a polydispersity index (PDI) of 0.115 ± 0.036, based on DLS measurements (Figure 1B). The monodisperse population of SPNs is confirmed by the moderate PDI and the SEM image in Figure 1C. The ζ-potential of SPNs was of −43.18 ± 9.23 mV, documenting the presence of negative surface charges associated with the carboxylic termination of the DSPE-PEG-COOH chains. The colloidal stability of SPNs was also assessed by measuring longitudinally, over a period of 9 days, both the hydrodynamic size and PDI. The resulting data (Figure 1D) document a remarkable stability of SPNs with a negligible size and PDI variation over the whole period.

Discoidal polymeric nanoconstructs were synthetized *via* a top-down fabrication approach combining lithographic techniques, template replications and polymer mixture loading, as previously described by the authors (14, 15). As shown in Figure 1E, DPNs appear as circular disks resulting from cross linking PLGA and poly(ethylene glycol) diacrylate (PEG-DA) chains. Following synthesis, DPNs were characterized for their physico-chemical properties. Specifically, DPN geometry (size and shape) was analyzed *via* Multisizer characterization and TEM. Size assessment of DPNs, measured in DI water *via* Multisizer, returned an average size of 787 ± 27 nm (Figure 1F). Given the non-sphericity of DPNs, their size spectrum cannot present a single, sharp peak as for SPNs in Figure 1B. The TEM image in Figure 1G confirms the discoidal shape with a diameter of ~1,000 nm and a height of ~ 400 nm. The ζ-potential of DPNs was around −19.9 ± 4.56 mV, resulting from the balance between the neutral charge of the PEG chains and the negative surface charge associated with the carboxylic termination on the PLGA chains. The colloidal stability of DPNs was also assessed by measuring longitudinally, over a period of 9 days, the average size *via* Multisizer. The data reported in Figure 1H document a remarkable stability over time even for this second nanoplatform.

### Analysis of Macrophage Interaction with SPNs and DPNs

**Figure 2:**
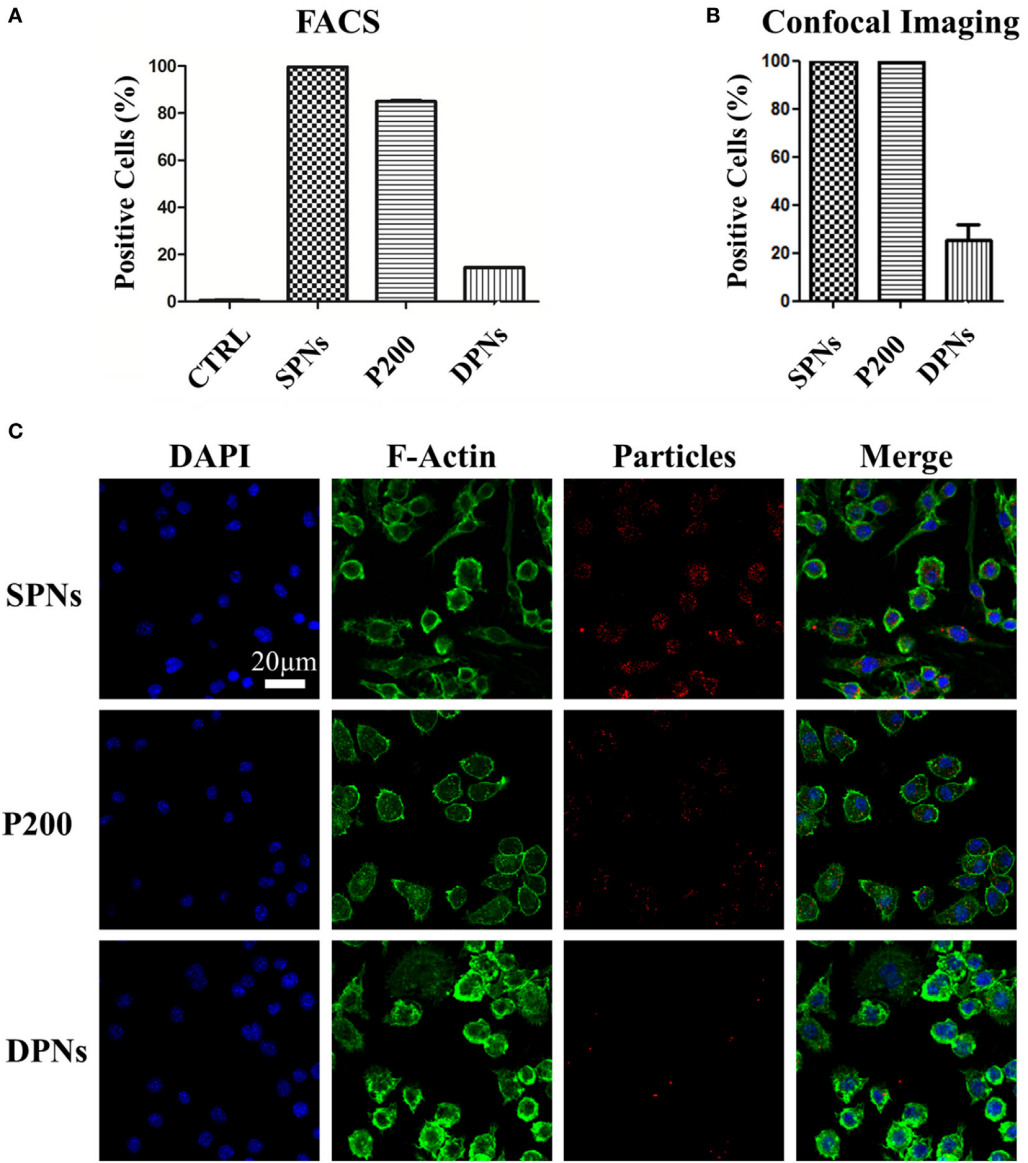
Macrophage association with spherical and discoidal polymeric nanoconstructs (SPNs and DPNs, respectively). **(A)** Cytofluorimetric analysis of RAW 264.7 cell association post 24-h incubation with SPNs, 200 nm polystyrene nanoparticles (P200) and DPNs (*n* = 3). **(B)** Confocal microscopy analysis of RAW 264.7 cell post 24-h incubation with SPNs, 200 nm polystyrene nanoparticles (P200) and DPNs (*n* = 4). **(C)** Representative confocal microscopy images of RAW 264.7 cells incubated with SPNs, P200, and DPNs (blue, DAPI staining for the nucleus; green, Alexa Fluor 488 Phalloidin staining for the cell cytoskeleton; red, SPNs, P200, and DPNs labeling with Rhodamine B). Scale bar: 20. µm.

Cytofluorimetric analysis was performed in order to assess SPN and DPN internalization into professional phagocytic cells. The same volume (~1 × 10^6^ μm^3^) of polymeric particles was used. Specifically, SPNs (1.5 µg/ml), DPNs (10 particles per cells), and 200 nm carboxylated polystyrene particles (P200) (30 particles per cells) were incubated with Raw 264.7 cells up to 24 h. At 24 h, the percentage of RAW 264.7 cells associated with particles was 99.6 ± 0.11% for SPNs, 84.9 ± 0.40% for P200, and 14.4 ± 0.0.06% for DPNs (Figure 2A). This trend was also confirmed *via* confocal microscopy analyses. Figure 2B shows that 100% of RAW 264.7 cells within a region of interest resulted to be positive to SPNs and P200, while only 25.3 ± 6.63% of macrophages turned to be associated with DPNs. The panel of Figure 2C shows representative microscopy images of RAW 264.7 cells 24-h post incubation with SPNs, P200 and DPNs. In Figure 2C, nuclei and actin filaments were stained in blue and green, respectively, whereas nanoparticles appeared as red dots. It is clearly confirmed the large difference in cell uptake between the spherical nanoparticles, SPNs and P200, and the discoidal nanoconstructs DPNs. Also, multiple SPNs and P200 are associated with the same cell.

### Pharmacological and Cytotoxicity Properties of SPNs

**Figure 3:**
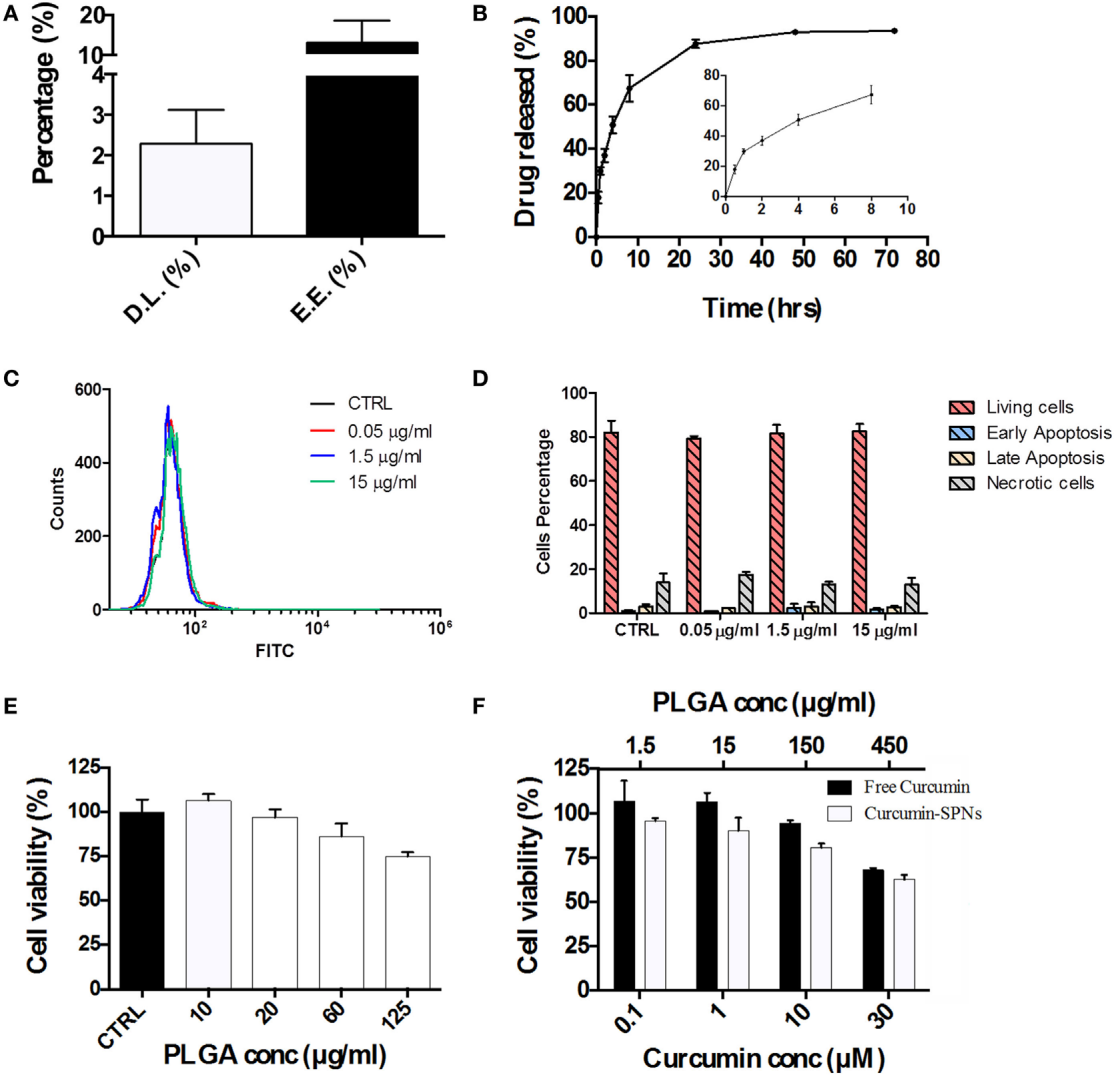
Pharmacological and cytotoxicity properties of SPNs and curcumin-loaded SPNs (Curc-SPNs). **(A)** Drug loading (DL) and encapsulation efficiency (EE) for Curc-SPNs (*n* = 3). **(B)** *In vitro* release profile of curcumin up to 72 h, under physiological conditions (pH 7.4 and 37°C). The inset shows the earlier time points of the curve (*n* = 3). **(C)** FITC fluorescent profile associated with Annexin V presence on cells membrane, after empty SPNs treatment or in control group. **(D)** Cells population distribution analysis on cells treated or not with nanoparticles, showing the level of viable, apoptotic, or necrotic cells. **(E)** Raw 264.7 cell viability at 24 h post incubation with empty SPNs (*n* = 5). **(F)** Raw 264.7 cell viability at 24 h post exposure to free Curcumin and Curc-SPNs (*n* = 5). Data are expressed as mean ± SD.

Based on the cell internalization results, SPNs were selected to be loaded with the natural, anti-inflammatory compound Curcumin. Given the hydrophobicity profile of this molecule, Curcumin was directly entrapped within the hydrophobic PLGA core of SPNs. Curc-SPNs present a hydrodynamic size of 193.4 ± 6.9 nm and a zeta potential of −43.8 ± 4.56 mV. The pharmacological properties of Curc-SPNs were characterized by quantifying DL and release, and cytotoxicity on RAW 264.7 macrophages. To this end, Curcumin EE and loading were assessed *via* spectrophotometric analysis. DL was calculated as the percentage in weight of loaded Curcumin compared to the total nanoparticle mass; whereas the EE was determined as the percentage of loaded Curcumin over the initial input amount of Curcumin. Data returned a DL of 2.31% ± 0.84 and an EE of 13.23% ± 5.41, as graphically reported in Figure 3A. The release profile of Curcumin was determined over a period of 72 h, under physiological conditions (pH = 7.4 and 37°C). As documented by the plot of Figure 3B, 50% of Curcumin was released within the first 6 h (see inset of Figure 3B), whereas the remaining 50% of anti-inflammatory molecules was slowly released within the following 66 h. The effect of SPNs in generating apoptosis, if any, was tested. Figure 3C shows the FITC intensity profiles, indicating the level of apoptosis, for all the experimental conditions used. There is no difference among cells untreated or treated with different empty SPNs concentrations, not even at a concentration three times higher than the one used for internalization experiments. Figure 3D shows more insights on the cell populations analyzed, showing the level of living cells, necrotic cells, or cells in early or late apoptosis. Also in this case, cells distribution in these subgroups is similar, despites the treatment used. Taken together, these results prove that our SPNs do not induce apoptosis, at least in the time frame and in the conditions used for the subsequent Curc-SPNs efficacy experiments. Finally, the cytotoxicity on Raw 264.7 cells of empty SPNs, free Curcumin, and Curc-SPNs was quantified at 24 h, using a MTT cell proliferation assay. Figure 3E shows the cell viability of Raw 264.7 incubated with empty SPNs. No significant toxicity of SPNs was detected up to more than 20 µg/ml of polymer. For larger concentrations, cell viability slightly reduces reaching an average value above 75% for 125 µg/ml of polymer, although without any significant difference with control. This is very important as this is the polymer concentration representing the amount of polymer of 10 µM Curc-SPNs. Figure 3F directly compares the cytotoxicity potential of free Curcumin and Curc-SPNs. No significant toxicity was detected up to 10 µM of Curcumin, whereas, for larger concentrations, cell viability reduced steadily in a concentration dependent fashion. Also, Curc-SPNs and free Curcumin returned comparable cytotoxicity activities on RAW 264.7.

### Anti-inflammatory Efficacy of Curc-SPNs

**Figure 4:**
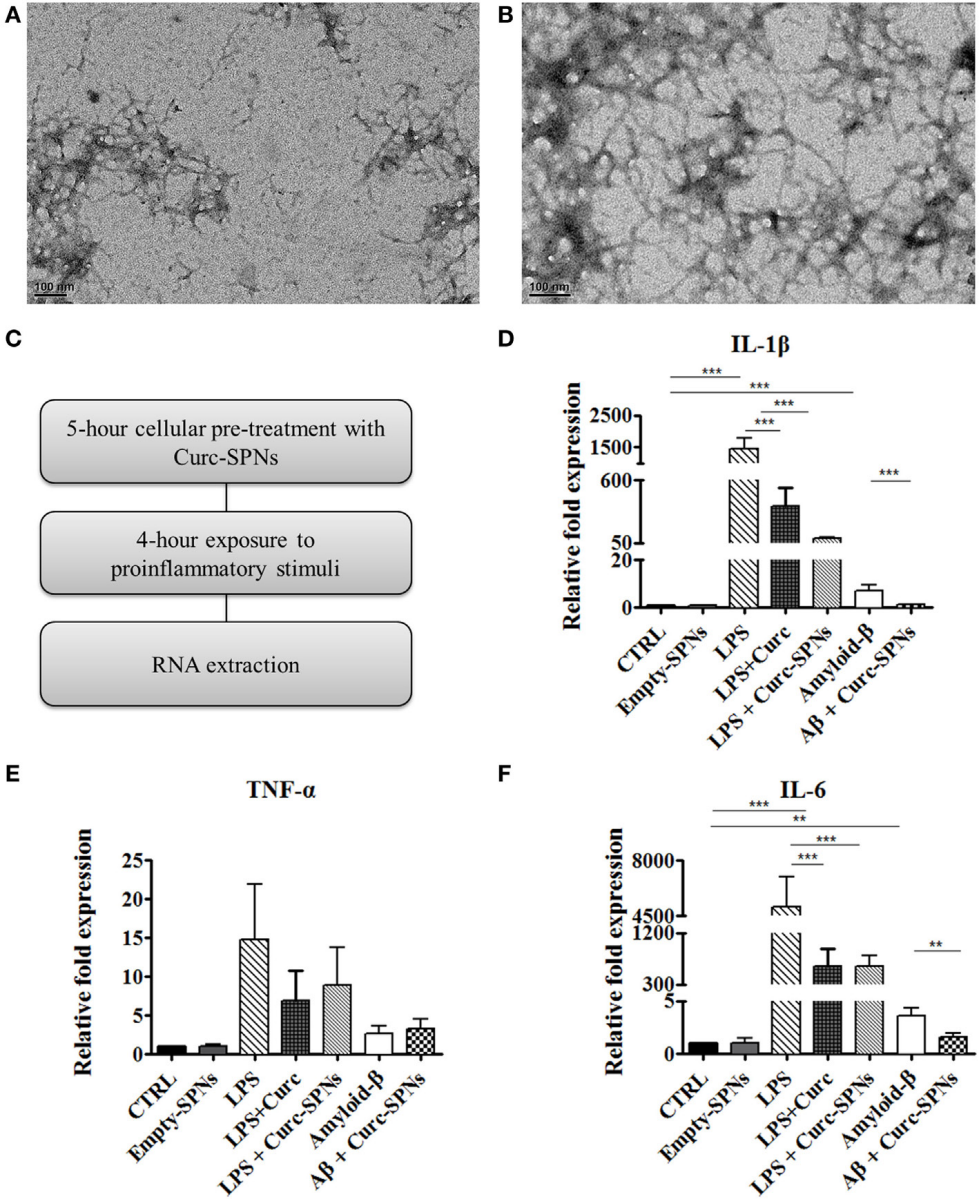
Mitigating cytokine production by Raw 264.7 macrophages. Transmission electron microscopy images of amyloid-β fibrils obtained by incubating Aβ (1–42) peptides at 37°C in 50 mM phosphate buffer (100 mM NaCl) for 6 h **(A)** and 16 h **(B)**. Following the experimental protocol showed in **(C)**, total RNA was collected and the mRNA levels of IL-1β **(D)**, TNF-α **(E)**, and IL-6 **(F)** were semi-quantified against GAPDH *via* Real Time RT-PCR (*n* = 3). Data are plotted as mean ± SD (“**” denotes statistically significant difference at *p* ≤ 0.01. “***” denotes statistically significant difference at *p* ≤ 0.005)).

Inflammation was induced in RAW 264.7 cells by two different methods: incubation with Aβ fibrils and incubation with LPS, as positive control. Fibrils were obtained through spontaneous polymerization by incubating Aβ (1–42) peptides at 37°C for 6 or 16 h. Both procedures yielded fibrillar structures of 6–9 nm in diameter and more than 200 nm in length, as shown in Figures 4A,B. Since no dramatic differences were observed between the two polymerization protocols, the 6-h polymerization was selected for all subsequent experiments.

For assessing the anti-inflammatory potential of Curc-SPNs, Raw 264.7 macrophages were pre-incubated for 5 h with Curc-SPNs (10 µM) and then exposed to an inflammatory stimulus for 6 h (2 µM of fibrillar Aβ or 100 ng/ml of LPS, as a positive control) (Figure 4C). The inflammatory response was assessed by measuring the levels of relevant pro-inflammatory cytokines, namely IL-1β, IL-6, and TNF-α, *via* RT-PCR (Figures 4D–F). No changes in gene expression were observed when treating cells with an equivalent dose empty SPNs, this additional control prove the presence of negligible levels of endotoxins, if any. Figures 4D–F show the relative fold expression levels for the three cytokines in six different experimental groups: the control group (CTRL)—cells were neither treated with SPNs not exposed to pro-inflammatory stimuli; the empty-SPNs group—cells were exposed to empty SPNs to verify the possible pro-inflammatory potential of nanoparticles; the LPS group—cells were exposed for 6 h to LPS without any SPN pre-treatment; the LPS + free Curcumin group—cells were pre-treated with 10 µM Curcumin and then stimulated by LPS; the LPS + Curc-SPNs group—cells were prior incubated with Curc-SPNs for 5 h and subsequently exposed to LPS for 6 h; the Amyloid-β group—cells were exposed for 6 h to fibrillar Aβ without any SPN pre-treatment; the Aβ + Curc-SPNs group—cells were prior incubated with Curc-SPNs for 5 h, and subsequently exposed to fibrillar Aβ for 6 h. Figure 4D shows that the cellular production of IL-1β is dramatically inhibited by a 5-h pre-treatment with Curc-SPNs. The relative cytokine expression reduces by ~15 times with respect to the case of LPS (from 1,439.67 ± 340.77 for LPS to 96.47 ± 4.33 for LPS + Curc-SPNs) and by ~6.5 times compared to fibrillar Aβ stimulation (from 7.21 ± 2.08 for Amyloid-β to 1.1 ± 0.17 for Aβ + Curc-SPNs). Free Curcumin treatment was also able to reduce IL-1β, although at a more modest level (369.28 ± 164.33) compared LPS stimulation. Similar observations can be drawn from Figure 4E for IL-6. The relative cytokine expression reduces by ~8 times when compared to the case of LPS stimulation (from 5,053.44 ± 1,928.49 for LPS to 618.19 ± 189.49 for LPS + Curc-SPNs) and by ~2.3 times to the case of fibrillar Aβ stimulation (from 3.61 ± 0.78 for Amyloid-β to 1.57 ± 0.41 for Aβ + Curc-SPNs). In this respect, free curcumin showed a very similar behavior (620.123 ± 302.51). Although the same general trend can also be depicted in Figure 4F, no statistically significant variations in the expression of TNF-α was observed for LPS and fibrillar Aβ stimulations, both in the case of free Curcumin and Curc-SPNs pre-treatment. This could be due to the specific phenotype of RAW 264.7 cells, stimulation times, and concentrations of pro-inflammatory stimuli.

## Discussion

Inflammation is relevant in the onset and progression of several diseases, including cancer, cardiovascular, metabolic, and neurodegenerative. Until the harmful stimulus persists, the inflammatory machinery evolves into a chronic process, reinforcing and turning itself into a injurious course, worsening the condition at the damaged site. Macrophages, phagocytic cells of myeloid origin, are key players in this kind of chronic inflammation (20). They can be found in every body tissue, assuming different phenotypes and roles: they can be differentiated into several kinds of cells, including microglia, Kupffer cells, and more. In this chronic insult scenario, macrophages proceed in their accumulation resulting in their continuous activation largely responsible for inducing damage. AD pathology is also characterized by an inflammatory response, which is primarily driven by the brain-resident macrophages, i.e., microglia, most likely toward Aβ plaques, exacerbating the pathology of the disease, escalating with its progression (5, 6). Microglia surrounds and is intimately associated with Aβ plaques, which in turn leads to the production of inflammatory cytokines and chemokines *in vitro* (21–28). Furthermore, a panoply of typical inflammatory mediators can be detected both in *in vivo* models and in brains or CSF from AD patients, including TNF-α, IL-1β, IL-6, GM-CSF, IL-12, and IL-23 (29–32). Even though microglia have the capacity to phagocyte Aβ, it is also true that the inefficient clearance of amyloid plaques is a major pathogenic factor in AD (33). Based on this, it is of crucial importance to try to modulate macrophages phenotype, inducing a regression in their pro-inflammatory activity. In this work, two different nanoconstructs—SPNs and DPNs—were presented and characterized with the objective of selecting the best nanoplatform for tempering inflammation in AD. Recently, different studies are trying to better understand and evaluate the interaction of nanoparticles and immune system cells. Small spherical nanoparticles, below 200 nm in diameter, seem to be more easily internalized by dendritic cells (34). Nonetheless, also nanoparticles’ softness and deformability plays a very important role in this process. In fact, both *in vitro* and *in vivo* data showed that harder particles are more prone to be phagocytized, as well as to be removed from blood circulation (e.g., through spleen filtration) (15, 35). Moreover, the comparison between SPNs and DPNs was not only instrumental to choose the best platform to be used, but also increased the knowledge of this special interaction, so useful for any nano-based drug delivery system. Cytofluorimetric analysis revealed that, at 24 h post incubation, 99.6% of RAW 264.7 cells were associated with SPNs as opposed to only 14.4% for DPNs. This behavior was also confirmed *via* confocal microscopy analysis. Notably, SPNs worked even better than polystyrene particles, chosen as a positive control since they are easily uptaken by phagocytic cells, as well documented in the literature (36, 37). The ability of SPNs to be internalized and modulate macrophages activity was already demonstrated by the authors (38–40). Therefore, these new data provide additional information, unequivocally suggesting that spherical nanoparticles are far better candidates for delivering anti-inflammatory drugs directly into macrophages compared to discoidal nanoconstructs. Consequently, SPNs were selected as the delivery platform and were loaded with Curcumin, a natural anti-inflammatory molecule, in order to deliver their payload inside target cells. Curcumin (diferuloylmethane) is a polyphenol that represents the major curcuminoid extracted from the *Curcuma longa* plant, which is extensively used for its anti-inflammatory, antioxidant, analgesic, antiseptic, and anticancer activity (41). In particular, its anti-inflammatory effect, mostly due to the inhibition of NF-kB transcription factor, makes Curcumin a highly desirable candidate as a therapeutic agent in several inflammation-based pathologies (42). However, Curcumin has a poor bioavailability of its hydrophobicity. For instance, in pre-clinical studies on rats, an oral dose of 500 mg/kg resulted in a peak plasma concentration of only 1.8 ng/ml (43). In a phase II clinical trial, 25 patients with pancreatic cancer were administered daily with 8 g of Curcumin leading to a maximum plasma level concentration of only 41 ng/ml (44, 45). In this manuscript, RAW 264.7 cells were exposed to amyloid-β fibrils and LPS, two potent pro-inflammatory stimuli, in order to replicate an inflamed environment and treated with free Curcumin and Curc-SPNs. Note that the stimulation of macrophages with Aβ fibrils was consider as a preliminary model of neuro-inflammation in AD (46). Results demonstrated a significant efficacy of Curc-SPNs in modulating the production of pro-inflammatory cytokines, namely IL-1β, IL-6, and TNF-α. Importantly, no changes in gene expression (in particular for IL-6 and TNF-α) (47) was observed upon the incubation of macrophages with empty SPNs. This additional control indirectly confirm the absence of endotoxin or of any unintentional contamination with minute amount of LPS, which would have induced a powerful inflammatory response (48). Interestingly, at the considered concentrations, the pro-inflammatory effect of LPS was always stronger than that associated with fibrillar Aβ incubation. Finally, no cytotoxic effect was observed after the incubation of the cells with the vehicle *per se*, as well as the ability of the system to preserve the structure and the function of Curcumin, after being loaded into the polymeric matrix. Altogether these findings demonstrate that spherical polymeric nanoconstructs can efficiently target macrophages and alleviate inflammation by the specific, intracellular delivery of Curcumin. This approach holds potential in the mitigation of inflammation in AD. However, future works should progress along two parallel paths. On the one hand, more sophisticated *in vitro* models will have to be considered where primary microglia and neurons are co-cultured and monitored over time during treatment for assessing cytokine production as well as neuronal activity. On the other hand, the role of tissue inflammation on the blood brain barrier permeability to nanoparticles should be elucidated using imaging and different pre-clinical models of AD.

## Author Contributions

AA: synthesis and chemico-physical and biological characterization of the spherical polymeric nanoconstructs (SPNs); RP: performed confocal microscopy and FACS analyses; AP: synthesis and chemico-physical and biological characterization of the discoidal polymeric nanoparticles (DPNs); AL and AC: helped with the synthesis and characterization of SPNs; DM: helped with the synthesis and characterization of SPNs and manuscript writing; PD: coordinated the work and manuscript writing. Results were analyzed and discussed by all authors.

## Conflict of Interest Statement

The authors declare that the research was conducted in the absence of any commercial or financial relationships that could be construed as a potential conflict of interest.
